# Smoking Dysregulates the Human Airway Basal Cell Transcriptome at COPD Risk Locus 19q13.2

**DOI:** 10.1371/journal.pone.0088051

**Published:** 2014-02-03

**Authors:** Dorothy M. Ryan, Thomas L. Vincent, Jacqueline Salit, Matthew S. Walters, Francisco Agosto-Perez, Renat Shaykhiev, Yael Strulovici-Barel, Robert J. Downey, Lauren J. Buro-Auriemma, Michelle R. Staudt, Neil R. Hackett, Jason G. Mezey, Ronald G. Crystal

**Affiliations:** 1 Department of Genetic Medicine, Weill Cornell Medical College, New York, New York, United States of America; 2 Department of Biological Statistics and Computational Biology, Cornell University, Ithaca, New York, United States of America; 3 Thoracic Service, Department of Surgery, Memorial Sloan-Kettering Cancer Center, New York, New York, United States of America; University of Tübingen, Germany

## Abstract

Genome-wide association studies (GWAS) and candidate gene studies have identified a number of risk loci associated with the smoking-related disease COPD, a disorder that originates in the airway epithelium. Since airway basal cell (BC) stem/progenitor cells exhibit the earliest abnormalities associated with smoking (hyperplasia, squamous metaplasia), we hypothesized that smoker BC have a dysregulated transcriptome, enriched, in part, at known GWAS/candidate gene loci. Massive parallel RNA sequencing was used to compare the transcriptome of BC purified from the airway epithelium of healthy nonsmokers (n = 10) and healthy smokers (n = 7). The chromosomal location of the differentially expressed genes was compared to loci identified by GWAS to confer risk for COPD. Smoker BC have 676 genes differentially expressed compared to nonsmoker BC, dominated by smoking up-regulation. Strikingly, 166 (25%) of these genes are located on chromosome 19, with 13 localized to 19q13.2 (p<10^−4^ compared to chance), including 4 genes (NFKBIB, LTBP4, EGLN2 and TGFB1) associated with risk for COPD. These observations provide the first direct connection between known genetic risks for smoking-related lung disease and airway BC, the population of lung cells that undergo the earliest changes associated with smoking.

## Introduction

Cigarette smoke, a major environmental stressor comprised of 10^14^ oxidants and >4000 chemicals in each puff, is the major cause of chronic obstructive pulmonary disease (COPD), a disease that originates in the airway epithelium, the cell population that takes the initial brunt of inhaled cigarette smoke [1]. However, only a fraction (∼20%) of smokers develop COPD, and some families have an increased risk to COPD, suggesting that host factors, likely inherited, modulate the risk for COPD from smoking [2]. Consistent with this concept, genome-wide association studies (GWAS), and candidate gene studies have identified COPD risk loci [3]–[5]. However, despite convincing evidence that inherited genetic variation conveys an increased risk of COPD in smokers, the relationship between these loci and the disordered biology of specific cell types within the lung is unclear.

As a strategy to begin to explore this association further, we have focused on airway basal cells (BC), the stem/progenitor cells capable of generating differentiated airway epithelium that comprises the continuous sheet of cells, including ciliated and secretory cells, covering the airways from the trachea to the terminal bronchioles [6], [7]. BC are the first airway cells to show abnormalities in response to smoking, including hyperplasia, altered differentiation and squamous metaplasia [8]. Stratified squamous basal cell epithelium is a recognized feature of COPD with increased differentiation of airway BC to mucous cell types [9]. Based on this knowledge, we hypothesized that BC may play a central role in genetic susceptibility to COPD and the early disordered lung biology associated with smoking.

Capitalizing on the ability to isolate BC from the airway epithelium of healthy individuals [6], we assessed whether smoking changes the transcriptional program of airway BC and whether this smoking-induced transcriptional dysregulation is relevant to the genetic susceptibility to smoking-related COPD. To accomplish this, we used massive parallel RNA-sequencing to compare the airway BC transcriptome of active smokers to that of life-long nonsmokers. The data not only demonstrates significant differences in the BC transcriptome of the active smoker compared to that of the nonsmoker, but interestingly, identified 13 genes dysregulated in the BC of smokers coded at chromosomal subband 19q13.2, a locus identified by GWAS [10] and candidate gene studies to confer risk for COPD (Table S1 in File S1). Notably, the expression of these 13 genes appears to be coordinately controlled in nonsmokers, but this coordinate control is partially lost in smokers, suggesting a multi-gene paradigm in the pathogenesis of COPD, in which clustered inheritance of multiple risk alleles, together with smoking-induced dissonant regulation of their expression, contributes to the early disordered biology of the airway epithelium that initiates the development of COPD. Together, these observations provide the first connection between a locus associated with risk for COPD and the dysregulation of airway basal cells, a cell population critical for normal airway structure and function, and central to the earliest histologic abnormalities associated with cigarette smoking.

## Methods

### Ethics Statement

All individuals were evaluated and samples collected in either the Weill Cornell NIH or the Rockefeller University Clinical and Translational Science Center and Department of Genetic Medicine Clinical Research Facility under clinical protocols approved by the Weill Cornell Medical College, Rockefeller University, and New York/Presbyterian Hospital Institutional Review Boards (IRB) according to local and national IRB guidelines. All subjects gave their informed written consent prior to any clinical evaluations or procedures.

### Human Airway Basal Cells

BC were isolated from the airway epithelium of healthy nonsmokers (n = 10) and healthy smokers (n = 7) as previously described [6]. All individuals had no significant past medical history, and physical examination, chest imaging and lung function was normal. There was no significant difference in age between nonsmokers and smokers, though nonsmokers tended to be younger. There was one female smoker; all other subjects were male. Smoking status was confirmed using urinary tobacco metabolites (Table S4 in File S1). BC were trypsinized and cytospin slides prepared for characterization by immunohistochemistry using cell-type specific markers (Supplemental Methods in file S1). All BC preparations were >95% positive for BC markers and negative for markers of other cell types [6].

### RNA Sequencing and Quantification of Gene Expression

Total RNA from harvested nonsmoker and smoker BC was extracted, mRNA libraries generated, RNA fragmented and cDNA synthesized as per protocol (Illumina, San Diego, CA). Purified ligation products were PCR amplified and resultant cDNA purified. Samples were loaded onto an Illumina flowcell for paired-end sequencing reactions using the Illumina HiSeq 2000 (Supplemental Methods in file S1).

Expression analysis was performed using Bowtie (v0.12.8.0), Tophat (v2.0.4) and Cufflinks (v2.0.2). To correct for transcript length and coverage depth, raw paired-end reads were converted into fragments per kilobase of exon per million fragments sequenced (FPKM). Resultant fragments were mapped to the reference genome build UCSC hg19 using Bowtie. Non-aligned reads were segmented using Tophat and re-aligned, thereby aligning reads that span introns and determining junction splice sites. Cufflinks assembled reads into transcripts and assembled reads were then merged using Cuffmerge (Supplemental Methods in file S1). Reads generated were directly proportional to transcript relative abundance.

To determine gene expression level above background, a false discovery rate (FDR) and false negative rate (FNR) were estimated by comparing the expression levels of known exons to intergenic regions (Figure S2 in File S1). The optimal expression value as defined by the intersection of the FDR and FNR was 0.04 FPKM. Genes with FPKM≥0.04 were scored as expressed. Partek Genomics Suite 6.6 (St. Louis, MO) was used to assess differential gene expression between nonsmokers and smokers. Notwithstanding small sample size, strict statistical criteria were employed to determine smoking-responsive genes using a cut-off in fold-change of 1.5 and adjusted p<0.05 with Partek “step-up” (Benjamini-Hochberg) FDR correction for multiple comparisons. Functional categories were assigned to the BC smoking signature using Affymetrix NetAffx Center, Human Protein Reference Database and GeneCards. Gene classification was performed using Ingenuity Pathway Analysis and gene set over-representation pathway analysis using ConsensusPath DB. The raw data and FPKM values are publically available at the Gene Expression Omnibus (GEO) site (http://www.ncbi.nlm.nih.gov/geo/), accession number GSE47718.

### Chromosomal Location of Airway BC Smoking-dysregulated Genes

To assess whether the smoker BC transcriptome was enriched with genes at or near GWAS single nucleotide polymorphisms (SNPs) for traits associated with smoking-induced COPD, a literature search was performed using search terms “smoking”, “candidate gene”, “genome wide association studies”, “GWAS”, “chronic obstructive pulmonary disease” and “COPD”. Search results were validated using the UCSC Genome Browser (http://genome.ucsc.edu/) and the NHGRI Catalog of Published GWAS Studies (www.genome.gov) determining the regions and specific genes identified by GWAS and candidate gene studies related to COPD phenotypes [3]–[5], [10]–[16]. Partek Genomics Suite was used to assign the BC smoking-dysregulated genes to chromosomal locations.

To assess the enrichment of smoking-dysregulated genes at chromosomal sites, the observed distribution across each site was compared to what could be expected by chance. 676 genes were randomly selected from all genes expressed above background after excluding the 676 smoking-responsive genes, and their respective chromosomal location recorded. This was repeated over 10,000 iterations, to obtain a null distribution, giving the expected chromosomal distribution of a randomly constructed gene set of equal size to that of our smoking-dysregulated gene list. Using the same approach, the enrichment of BC smoking-dysregulated genes was also assessed in COPD GWAS loci at the chromosome and chromosome subband levels. All analysis was performed using R version 2.15.1 statistical software.

### Assessment of Coordinate Control

To assess coordinate control of the 13 BC smoking dysregulated genes localized to subband 19q13.2, a correlation matrix was constructed by computing the Pearson correlation coefficient measure between all pairs of genes belonging to the 13 gene sets. Pearson correlation coefficients were computed using statistical software R version 2.15.1 separately for nonsmoker and smoker BC gene expression.

### Copy Number Variation and Methylation Influences on 19q13.2 Airway Epithelium Gene Expression

To assess possible mechanisms of why smoking is associated with up-regulation of genes localized to 19q13.2, we asked: (1) could the study population of smokers have copy number variations (amplification) or the nonsmokers copy number variations (deletions) in this region; (2) could smoking modulate airway DNA methylation in this region?

Copy number variation analysis of blood DNA was performed using Partek Genomics Suite segmentation analysis with a minimum of 10 probes, first on 85 Affymetrix Genome-Wide SNP 6.0 microarrays of an independent cohort of 23 healthy nonsmokers and 62 healthy smokers and then on 6 nonsmokers and 6 smokers from the basal cell study population. To assess possible smoking-related methylation changes in airway epithelial DNA in the region 19q13.2, DNA from complete airway epithelium of 19 nonsmokers and 20 smokers was assessed by the HELP assay for the methylation status of 117,521 HpaII fragments as previously described [17].

### Assessment of the Complete Airway Epithelium Expression of 19q13.2 Basal Cell Smoking Dysregulated Genes

Although BC represent only a minority of the total airway epithelium, we assessed gene expression microarrays of the total airway epithelium to see if a similar signal of 19q13.2-relevant smoking-related gene expression might be detected in the complete epithelium. To accomplish this, we used Affymetrix U133 Plus 2.0 microarray of airway epithelium of smokers (n = 31) *vs* nonsmokers (n = 21) of the same order of bronchi of airway epithelium from which the nonsmoker and smoker BC were derived.

## Results

### Effect of Smoking on the Airway BC Transcriptome

**Figure 1:**
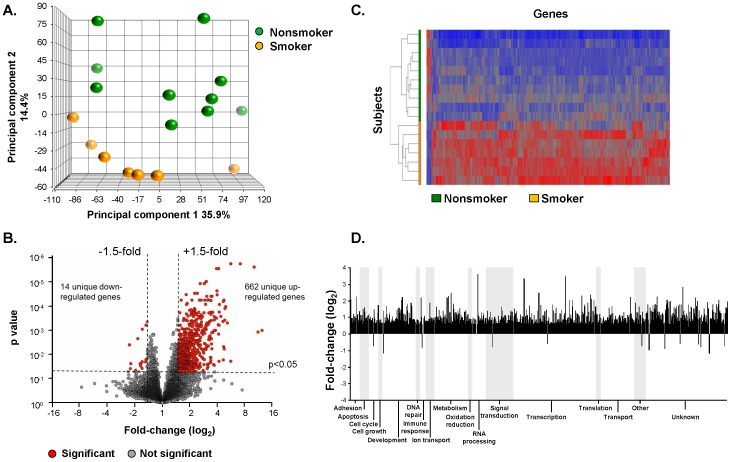
Smoking-induced dysregulated transcripts in human airway basal cells. **A.** Principal component analysis. Shown is gene expression of basal cells (BC) of nonsmokers (n = 10, green circles) and smokers (n = 7, orange circles) using all 13,385 expressed genes as an input dataset. **B.** Volcano plot, smoker *vs* nonsmoker airway BC. Ordinate – p value; abscissa – fold-change (log_2_). **C.** Hierarchical cluster analysis of smoker *vs* nonsmoker basal cells based on expression of 676 smoking-dysregulated genes [fold-change>1.5, p<0.05 with false discovery rate (FDR) correction]. Genes expressed above the average are represented in red, below average in blue and average in grey. The genes are represented vertically, and individual samples horizontally. **D.** Functional categories of the 676 unique genes significantly differentially expressed in smoker *vs* nonsmoker human airway BC (≥1.5 fold-change up- or down-regulated; p<0.05 with FDR correction). Shown are fold-changes of the smoking-responsive genes on a log_2_ scale.

A total of 13,385 RefSeq annotated genes were expressed above background in nonsmoker and smoker BC. Average gene expression across all subjects was 32.2 FPKM, with no significant difference between smokers and nonsmokers (p>0.05). Principal component analysis, using all expressed genes as an input dataset, demonstrated clear separation of samples by smoking phenotype (Figure 1A). Altered gene expression in smoker BC could result, in part, from the culture conditions; however, identical culture conditions were used to culture the BC from nonsmokers. A volcano plot identified 662 significantly up-regulated genes and 14 significantly down-regulated genes using criteria of fold-change >1.5 and adjusted p<0.05 with Partek “step-up” (Benjamini-Hochberg) FDR correction for multiple comparisons (Figure 1B). Unsupervised hierarchical cluster analysis using the 676 smoking-dysregulated gene list revealed complete separation of smoker and nonsmoker BC gene expression (Figure 1C). The dominant categories enriched among the BC smoking-dysregulated genes included development, metabolism, signal transduction and transcription (Figure 1D).

Among the top 50 BC smoking-dysregulated genes, ordered by absolute difference in gene expression, were several related to oxidative stress, including glutathione peroxidase (GPX1) which was up-regulated, and microsomal glutathione S-transferase 1 (MGST1), which was one of the few genes down-regulated by smoking (Table 1). The most common functional categories in the top 50 BC smoking dysregulated genes were those associated with transcription (14/50, 28%), development (7/50, 14%), apoptosis (6/50, 12%) and signal transduction (5/50, 10%; Table 1). Other categories included genes relevant to interactions with the extracellular matrix (adhesion, cytoskeleton and extracellular matrix), calcium ion channels (Table S2 in File S1) and genes encoding central components of the signaling pathways previously shown to be enriched in the airway BC transcriptome [6], such as NF-κB, vascular endothelial growth factor (VEGF), epidermal growth factor receptor (EGFR), Notch, and transforming growth factor beta (TGF-β); (Figure S1 in File S1). Pathway analysis identified overrepresentation of pathways with known relevance to airway BC stem/progenitor cells [6], [18], [19], including integrin, Notch and EGFR pathways (Table S3 in File S1).

**Table 1:** Top 50 Smoking-dysregulated Genes in Human Airway Basal Cells^1^.

Functional category	Gene symbol	Gene title	Nonsmoker mean expression level (FPKM)^2^	Smoker mean expression level (FPKM)^2^	Absolute Difference^3^	Fold-change^4^	p value^5^
Adhesion	ITGB4	integrin, beta 4	233.5	375.7	142.2	1.6	3.2×10^−2^
	COL7A1	collagen, type VII, alpha 1	63.2	122.6	59.4	1.9	2.6×10^−2^
	PKP3	plakophilin 3	38.7	74.4	35.7	1.9	6.0×10^−3^
Apoptosis	TNFRSF12A	tumor necrosis factor receptor superfamily, member 12A	152.8	267.1	114.3	1.7	1.2×10^−2^
	ARHGDIA	Rho GDP dissociation inhibitor (GDI) alpha	103.4	185.7	82.3	1.8	1.5×10^−2^
	PHLDA2	pleckstrin homology-like domain, family A, member 2	34.1	104.3	70.2	3.1	1.0×10^−3^
	FKBP8	FK506 binding protein 8, 38 kDa	70.1	106.8	36.7	1.5	3.5×10^−2^
	MFSD10	major facilitator superfamily domain containing 10	30.0	65.3	35.3	2.2	2.0×10^−3^
	BAD	BCL2-associated agonist of cell death	20.3	52.4	32.2	2.6	3.0×10^−4^
Development	PPDPF	pancreatic progenitor cell differentiation and proliferation factor homolog (zebrafish)	159.8	385.9	226.1	2.4	7.0×10^−3^
	PLEC	plectin	69.5	186.3	116.8	2.7	1.0×10^−3^
	JUNB	jun B proto-oncogene	44.6	134.2	89.5	3.0	4.0×10^−3^
	FSCN1	fascin homolog 1, actin-bundling protein (Strongylocentrotus purpuratus)	107.4	186.1	78.7	1.7	4.2×10^−2^
	ARPC1B	actin related protein 2/3 complex, subunit 1B, 41 kDa	114.8	180.7	65.9	1.6	1.2×10^−2^
	LAMA5	laminin, alpha 5	19.7	54.6	34.8	2.8	2.0×10^−3^
	BCAR1	breast cancer anti-estrogen resistance 1	14.0	45.3	31.3	3.2	1.9×10^−4^
Immune response	PDLIM7	PDZ and LIM domain 7 (enigma)	35.9	73.6	37.7	2.1	2.0×10^−3^
Ion transport	SLC16A3	solute carrier family 16, member 3 (monocarboxylic acid transporter 4)	82.7	142.5	59.8	1.7	1.9×10^−2^
	GLTSCR2	glioma tumor suppressor candidate region gene 2	44.0	93.2	49.1	2.1	7.0×10^−2^
Metabolism	MIF	macrophage migration inhibitory factor (glycosylation-inhibiting factor)	32.6	182.6	150.0	5.6	1.8×10^−6^
	MZT2B	mitotic spindle organizing protein 2B	23.7	84.3	60.6	3.6	1.8×10^−4^
	CHPF	chondroitin polymerizing factor	25.6	67.3	41.7	2.6	2.7×10^−4^
	NAPRT1	nicotinate phosphoribosyltransferase domain containing 1	17.4	49.5	32.1	2.8	2.6×10^−2^
Oxidation reduction	GPX1	glutathione peroxidase 1	243.0	464.3	221.4	1.9	1.4×10^−2^
Signal transduction	GPC1	glypican 1	42.4	86.0	43.6	2.0	2.1×10^−2^
	DVL1	dishevelled, dsh homolog 1 (Drosophila)	25.5	62.1	36.6	2.4	1.0×10^−3^
	MAP7D1	MAP7 domain containing 1	38.7	70.2	31.5	1.8	2.7×10^−2^
	LRP10	low density lipoprotein receptor-related protein 10	56.1	87.3	31.2	1.6	2.2×10^−2^
	IER2	immediate early response 2	38.0	68.6	30.6	1.8	8.0×10^−3^
Transcription	TSPO	translocator protein (18 kDa)	92.7	220.8	128.1	2.4	1.0×10^−3^
	MGST1	microsomal glutathione S-transferase 1	358.2	236.4	121.8	−1.5	1.0×10^−3^
	AGRN	agrin	40.9	116.6	75.7	2.9	1.9×10^−4^
	RNH1	ribonuclease/angiogenin inhibitor 1	76.0	150.0	73.9	2.0	2.8×10^−2^
	CEBPD	CCAAT/enhancer binding protein (C/EBP), delta	25.4	71.0	45.6	2.8	4.6×10^−2^
	GIPC1	GIPC PDZ domain containing family, member 1	62.7	104.4	41.8	1.7	5.0×10^−3^
	EPN1	epsin 1	29.7	70.3	40.6	2.4	3.6×10^−4^
	H1FX	H1 histone family, member X	24.4	64.1	39.7	2.6	2.0×10^−3^
	ADRM1	adhesion regulating molecule 1	51.6	85.6	34.0	1.7	1.3×10^−2^
	IRAK1	interleukin-1 receptor-associated kinase 1	50.8	84.4	33.7	1.7	2.6×10^−2^
	NDUFS7	NADH dehydrogenase (ubiquinone) Fe-S protein 7, 20 kDa (NADH-coenzyme Q reductase)	19.1	51.2	32.1	2.7	1.0×10^−3^
	HOMER3	homer homolog 3 (Drosophila)	35.6	67.7	32.1	1.9	3.0×10^−3^
	RBCK1	RanBP-type and C3HC4-type zinc finger containing 1	26.5	55.5	29.0	2.1	3.5×10^−2^
	CEBPB	CCAAT/enhancer binding protein (C/EBP), beta	26.4	55.3	28.9	2.1	2.1×10^−2^
Translation	RPLP1	ribosomal protein, large, P1	1632.0	2533.0	901.0	1.6	4.8×10^−2^
	MRPL41	mitochondrial ribosomal protein L41	22.5	71.0	48.5	3.2	1.0×10^−3^
Other	ATP6V0C	ATPase, H+ transporting, lysosomal 16 kDa, V0 subunit c	74.9	138.6	63.7	1.9	1.0×10^−3^
Unknown	MSMO1	methylsterol monooxygenase 1	207.6	119.7	87.9	−1.7	3.0×10^−2^
	CCDC124	coiled-coil domain containing 124	30.4	69.4	39.1	2.3	2.4×10^−6^
	MZT2A	mitotic spindle organizing protein 2A	25.6	56.8	31.2	2.2	2.4×10^−2^
	ZNF598	zinc finger protein 598	14.8	44.5	29.6	3.0	6.0×10^−3^

1 Top 50 genes identified by largest absolute difference in expression and sorted in descending order of absolute difference within each category.

2 FPKM = fragments per kilobase of exon per million fragments mapped.

3 Absolute difference = smoker mean - nonsmoker mean.

4 Fold-change = mean in smokers/mean in nonsmokers.

5 False discovery rate controlled to 0.05 using Partek ‘step-up’ (Benjamini-Hochberg) procedure.

### Genetic Variation and BC Smoking-responsive Genes

**Figure 2:**
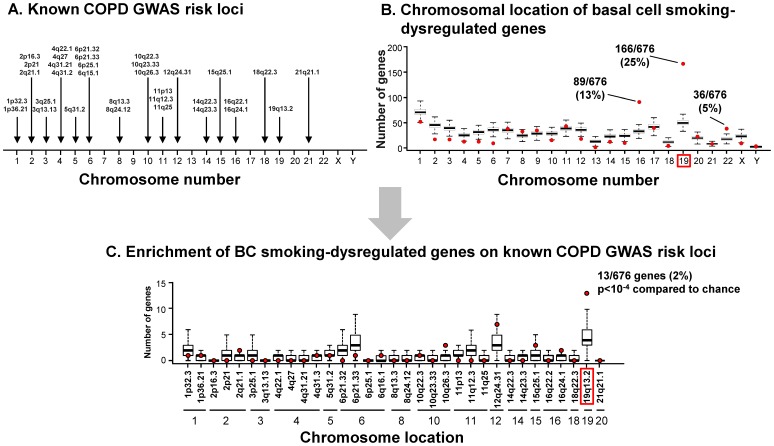
Comparison of chromosomal location of basal cell smoking-dysregulated genes to known COPD risk loci. **A.** Chromosomal distribution of SNPs identified by GWAS (p<10^−5^) as risk loci for COPD and related phenotypes. **B.** Chromosomal location of the 676 significant smoking-dysregulated basal cell (BC) genes as compared to distribution expected by random chance. Red dots represent the number of BC smoking-dysregulated genes localized to each chromosome. Box and whisker plots represent 10^4^ permutations of 676 randomly chosen genes. The red dots above chromosomes 16, 19 and 22 represent the number of BC smoking-dysregulated genes in each location, along with the % of total dysregulated genes on each chromosome. **C.** Enrichment of 676 BC smoking-dysregulated genes on known COPD risk loci. Red dots represent number of BC smoking-dysregulated genes at each known COPD risk loci; the loci are identified by chromosome number and chromosome subband. Box and whisker plots represent the distribution of 676 randomly chosen genes permutated 10^4^ times for each GWAS chromosome subband location. The only statistically significant locus was 19q13.2 (p<10^−4^).

**Figure 3:**
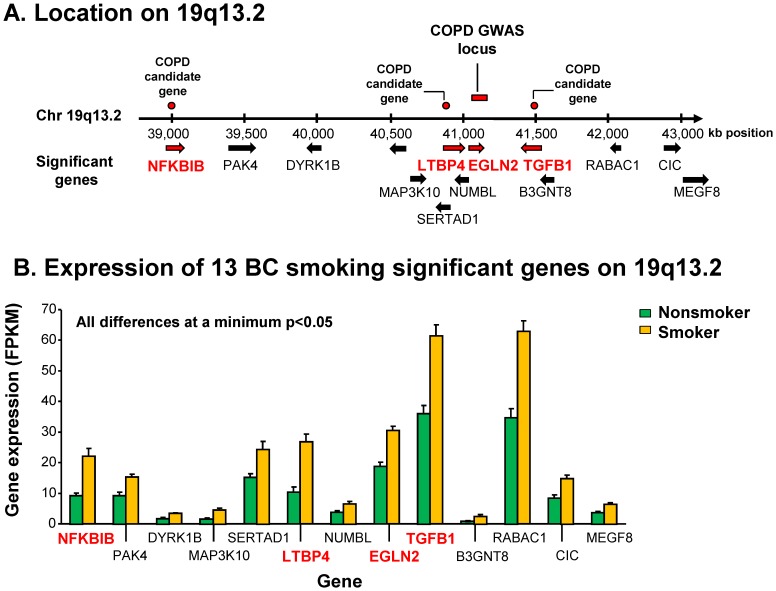
Basal cell (BC) smoking-dysregulated genes localized to COPD-risk locus 19q13.2. **A.** Genome distribution of 13 significant BC smoking-dysregulated genes on locus 19q13.2. Red bar – known COPD locus; red dots – known COPD candidate genes (see Table S1 in File S1). **B.** Expression of BC smoking-dysregulated genes on 19q13.2. Expression is in fragments per kilobase of exon per million fragments mapped (FPKM). Nonsmoker (n = 10, green bars), smoker (n = 7, yellow bars). All smoker to nonsmoker comparisons minimum p<0.05. The 4 COPD risk genes are in red.

The chromosomal distribution of the 676 smoking-dysregulated genes was mapped to the chromosomal distribution of the COPD risk alleles as compared to random chance accounting for gene density per region (Figure 2A, B). This analysis revealed statistically significant enrichment of BC smoking-dysregulated genes (291/676; 43%; p<10^−4^) on chromosomes 16, 19 and 22, with 13% (89 of 676) on chromosome 16, 5% (36/676) on chromosome 22 and 25% (166/676) on chromosome 19, a locus that was first identified as a COPD risk locus by genetic linkage analysis (Table S1 in File S1). Strikingly, however, 13 of 676 (2%) BC smoking-dysregulated genes were significantly localized to chromosome subband 19q13.2 (p<10^−4^, Figure 2C), including NFKBIB, PAK4, DYRK1B, MAP3K10, SERTAD1, LTBP4, NUMBL, EGLN2, TGFB1, B3GNT8, RABAC1, CIC and MEGF8 (Figure 3A). All of these genes were up-regulated in smokers, although the extent to which each gene was upregulated varied considerably (Figure 3B). Among the most up-regulated were NFKBIB, LTBP4, EGLN2, and TGFB1, all of which have been previously associated with an increased risk for COPD in GWAS and/or candidate gene studies (Table S1 in File S1), and EGLN2 has been clearly identified at a risk locus by a recent GWAS publication [10].

**Figure 4:**
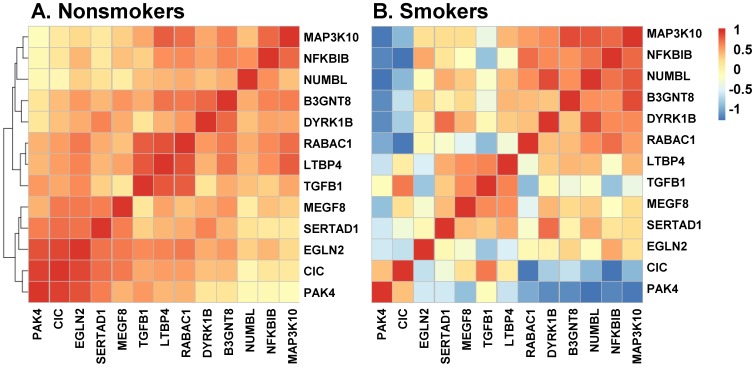
Hierarchical clustering of the correlation coefficients of mean gene expression of 13 smoking-dysregulated genes on chromosome locus 19q13.2 in nonsmoker and smoker BC. **A.** Nonsmokers; **B.** Smokers. The correlation coefficients allow us to assess the relationship between pairs of genes, and range from −1 (blue) to 1 (red). Positive correlation coefficient is represented in red, consistent with co-expression in the same direction. Negative correlation coefficient is represented in blue, consistent with co-expression in opposite directions.

Comparison of the levels of expression of these 13 genes in BC of nonsmokers revealed a significant correlation, suggesting the possibility that in nonsmokers, the expression of these genes in BC is coordinately controlled (r^2^ = 0.58, p<0.025; Figure 4A). Additionally, clusters of high correlation coefficients were observed between the PAK4-CIC-EGLN2 triplet (r^2^ = 0.92, p<0.05), the TGFB1-LTPB4-RABAC1 triplet (r^2^ = 0.88, p<0.05) and the NFKBIB-MAP3K10 couple (r^2^ = 0.83; p<0.05). Interestingly, although a subset of genes (MAP3K10, NFKBIB, NUMBL and B3GNT8), maintained coordinate control in smokers (r^2^ = 0.80, p<10^−3^), the overall mean coordinate control of the 13 BC smoking-dysregulated genes in smokers was lost (mean r^2^ = 0.48 in smokers; p = 0.26) compared to what would be expected by chance (Figure 4B).

### Possible Mechanisms Underlying the Concentration of Smoking Up-regulation of Genes at the 19q13.2 Locus

Two levels of control were evaluated as possible mechanisms of the concentration of smoking up-regulated genes at 19q13.2, including: (1) CNV duplication of genes at 19q13.2; and (2) smoking-related methylation changes of airway epithelial DNA in the 19q13.2 region. For both of these assessments, we used nonsmoker and smoker cohorts independent of the cohorts used for the BC smoking transcriptome analysis.

CNV analysis did not demonstrate changes that could explain the concentration of smoking up-regulated genes at 19q13.2. CNV analysis of blood DNA of an independent cohort of 23 healthy nonsmokers and 62 healthy smokers revealed no CNVs in the 19q13.2 region. Further, CNV analysis of 6 smoker and 6 nonsmoker BC subjects in the BC transcriptome analysis revealed no CNVs in this region.

Likewise, assessment of smoking-related airway epithelium DNA methylation changes did not show differences relevant to 19q13.2. Comparison of DNA methylation patterns between 19 healthy nonsmokers and 20 healthy smokers revealed 204 differentially methylated genes [17]. There were 2 airway epithelium genes hypermethylated in smokers as compared to nonsmokers on 19q13.2 (CYP2F1 and RASGRP4), neither of which were significantly differentially regulated by smoking in airway BC.

We also assessed microarray analysis of the transcriptomes of the complete airway epithelium of smokers *vs* nonsmokers to see if the BC smoking dysregulated genes could be observed even in the context that the BC only represent a small minority (15 to 20%) of the cell population [20]. Analysis of Affymetrix U133 Plus 2.0 microarray, was carried out in airway epithelium of the same order bronchi as the BC of smokers (n = 31) *vs* nonsmokers (n = 21). However, as expected because of the minority representation of BC in the complete airway epithelium, of the 4 smoking BC dysregulated genes localized to 19q13.2 that have been identified as a COPD or smoking-related genes (either GWAS or candidate; NFKBIB, LTBP4, EGLN2, TGFB1), none were significantly different between nonsmokers and smokers. In addition, the smoker BC gene clusters at specific chromosome loci were not a feature of the smoker complete airway epithelium, consistent with prior data showing distinct nonsmoker BC compared to the complete airway epithelium transcriptomes, consistent with knowledge that BC make up only a small percentage of cells comprising the complete airway epithelium [6].

## Discussion

While there is overwhelming evidence that cigarette smoking is the major cause of COPD, it is also clear that only a fraction of smokers develop disease, suggesting that inherited genetic variation modulates susceptibility to the development of COPD [2]. Consistent with this concept, GWAS and candidate genes studies together have made a convincing case that genetic variability plays an important role in conveying risk for COPD [3]–[5], [10]–[16]. However, like most complex human disorders, while the observed loci are clearly associated with disease risk, the relationship of these loci/genes with disease pathogenesis is unclear.

Based on the knowledge that airway BC function as the stem/progenitor cells of the differentiated airway epithelium [6], [7] and that BC hyperplasia is an early pathologic lesion associated with smoking, followed by disordered airway epithelial differentiation and squamous metaplasia [8], we hypothesized that the smoking-related disordered biology of airway BC and the early pathologic lesions associated with smoking could have genetic origins at COPD risk loci, thereby implicating airway BC in the pathogenesis of smoking-related COPD. Despite the potential limitation of small sample size, the data strikingly demonstrates that smoking significantly alters the transcriptional program of airway BC, with marked dysregulation of 676 genes compared to that of BC of nonsmokers. Unexpectedly, we found that 25% of these 676 dysregulated genes were localized to chromosome 19, with 13/676 (2%) of these genes on locus 19q13.2, an observation that far exceeded random chance. Interestingly, subband 19q13.2 is the same region where GWAS and candidate gene studies have identified SNPs associated with a risk for COPD (Table S1 in File S1) and for smoking behavior [21], [22]. Together, these observations relate the genetic variability-associated risk for COPD to the cell population that exhibits the earliest pathologic lesions associated with pathogenesis of cigarette smoking-induced COPD.

### BC Smoking-dysregulated Genes on 19q13.2 and COPD Risk

Sequence variations of chromosome 19, and in particular subband 19q13.2, have been implicated in a number of GWAS and candidate gene studies as conveying a risk to COPD in relation to smoking (Table S1 in File S1). Of the 13 BC smoking-dysregulated genes localized to 19q13.2, four, NFKBIB, LTBP4, EGLN2 and TGFB1, have been implicated by GWAS and/or candidate gene studies to be a risk for developing COPD.

TGFB1 (transforming growth factor beta 1) is a multifunctional growth factor that affects a number of biological processes relevant to the pathogenesis of COPD. In agreement with our data that BC from smokers express increased levels of TGFB1, smoking promotes airway TGF-beta expression in association with collagen deposition in animal models [23]. Epithelial expression of TGF-beta in the lungs of COPD patients correlates with the decrease of forced expiratory volume in 1 second (FEV1), the hallmark of airway obstruction [24]. TGF-beta is generally secreted as a part of a latent complex, which includes the growth factor, its propeptide, and latent TGF-beta binding protein (LTBP), with LTBP4 specifically binding to only TGF-beta 1 [25]. Expression of LTBP4 is critical for the development and maintenance of lung architecture, LTBP4 variants are associated with impaired alveolarization and airway collapse [26], and LTBP4 null mice develop emphysema [27]. It is remarkable that both TGF-beta and LTBP4 are found up-regulated in the airway BC of smokers in the present study and that polymorphisms in genes encoding both TGF-beta and LTBP4 genes are associated with COPD susceptibility (Table S1 in File S1).

EGLN2 (Egl nine homolog 2), also known as prolyl hydroxylase domain-containing protein 1 (PHD1), is a cellular oxygen sensor [28], [29]. It is one of three isoforms that target the hypoxia inducible factor 1 alpha (HIF1α) transcriptional complex for degradation in response to hypoxia [29], with HIF1α degradation implicated in emphysema pathogenesis through VEGF pathways [30]. Through its effects on HIF1α, EGLN2 could influence >100 hypoxia-inducible target genes involved in cell proliferation/apoptosis, VEGF signaling and carbohydrate metabolism [29]. EGLN2 has been associated with COPD risk by a recent GWAS study [10]. Relevant to the disordered epithelium in COPD, EGLN2 increases cell proliferation, mediated by regulation of cyclin D [31] and may represent a mechanism by which smoking induces BC hyperplasia. Moreover, increased EGLN2 expression is associated with impaired epithelial junctional barrier function leading to increased epithelial permeability [32], which is a characteristic of the airway epithelium of healthy and COPD smokers [18]. EGLN2 regulates activity of NF-κB, a key transcriptional factor involved in activation of inflammatory and immune genes, including those implicated in COPD pathogenesis [33]. Notably, NFKBIB (NF-kappa-B inhibitor beta) is another COPD risk-associated gene in the 19q13.2 locus up-regulated in BC of smokers. Based on the knowledge that one of the functions of NFKBIB is to stabilize NF-κB responses [34], it is possible that up-regulation of this gene in airway BC plays a role in regulation of inflammatory responses in the smoker airways. Moreover, it has been shown that NFKBIB is part of cigarette smoke-induced oxidative stress response mediated via nuclear factor erythroid 2-related factor (NRF2) relevant to the pathogenesis of smoking-induced COPD [35].

### Other BC Smoking-dysregulated Genes on 19q13.2

Although nine of the 13 significant BC smoking dysregulated genes localized to 19q13.2 have not been specifically identified as COPD risk alleles, all are in the region of the COPD risk locus, and each has properties relevant to COPD pathogenesis. PAK4 (serine/threonine-protein kinase) regulates cell morphology, cytoskeletal organization, cell proliferation and migration, has anti-apoptotic functions [36] and is required for normal apical junction formation in human bronchial epithelium [37]. PAK4 protects the lung against oxidative stress [38], and PAK4 overexpression with activation of the pro-survival Akt pathway could represent an alternate pathway to smoking-induced BC hyperplasia [38]. DYRK1B (dual-specificity tyrosine phosphorylation-regulated kinase 1B) is a member of the evolutionarily conserved family of DYRK protein kinases with key roles in the control of cell proliferation and differentiation [39]. MAP3K10 (mitogen-activated protein 3 kinase 10), like PAK4 and DYRK1B, is a human epithelial serine threonine kinase. The main function of MAP3K10 is activation of JUN signaling and, using this mechanism, MAP3K10 regulates cell proliferation and apoptosis [40]. SERTAD1 (SERTA domain-containing protein 1) is a transcription factor that regulates the cell cycle, and known to bind prolyl hydroxylase motifs [41]. Overexpression of SERTAD1 induces genomic instability in cancer cell lines [42] and inhibits oxidant-induced cell death [43]. NUMBL (numb-like) encodes a cytoplasmic protein involved in Notch and NF-κB signaling relevant to stem cell self-renewal and differentiation [44], [45]. Overexpression of NUMBL has been associated with carcinogenesis and correlates with poor survival in metastatic non-small cell lung cancer [46]. B3GNT8 (β1,3-N-acetylglucosaminyltransferase) plays a role in carbohydrate metabolism, is expressed in the lung and up-regulated in epithelial cancers [47]. RABAC1 (phenylated Rab acceptor protein 1) encodes an integral membrane protein which strongly binds the nearby gene RAB4B on 19q13.2 [48]. Notably, EGLN2 and RABAC1 together form part of a 4-gene signature of invasive lung cancer [49]. CIC (protein capicua homolog) is a member of the HMG-box superfamily of transcription factors and modulates c-erb signaling via transcriptional repression [50]. As a broad regulator of receptor tyrosine kinase signaling, CIC plays an important role in the control of cell proliferation, survival and differentiation [51]. MEGF8 (multiple EGF-like domain containing 8) encodes a membrane associated protein with EGF-like domains. Although specific functions of MEGF8 are unclear, EGF and other molecules with EGF-like domains, such as mucins, are relevant to COPD pathogenesis [52]. EGFR signaling is enriched in the human airway BC transcriptome and smoking activates EGFR and related pathways in human airway BC [6]. Induction of MEGF8 in airway BC may interrupt adherens junction formation in smoker BC with effects on structural integrity of the airway epithelium [52].

### Airway BC-centered, Multi-gene Paradigm of COPD Pathogenesis

What are the possible explanations for smoking-related BC dysregulation of genes concentrated at 19q13.2? Based on the knowledge that >99% of all cells of the complete differentiated airway epithelium are derived from BC, we assessed this question by examining the airway epithelium of independent cohorts of nonsmokers and smokers for 2 possible explanations: (1) CNV duplications at 19q13.2; and (2) smoking-related methylation changes of airway epithelium DNA at 19q13.2. The data assessing CNVs and methylation changes showed no relation to 19q13.2. Thus, at least for now, the mechanism underlying the concentrated dysregulation of smoking-related BC genes is not understood. The 19q13 locus has been associated with smoking behavior and more recently with COPD [10], [21], [22]. Thus, as the subjects in this study are healthy smokers who may or may not develop COPD, it is unclear whether the finding of gene clusters at locus 19q13.2 is a smoking and/or a COPD associated relationship. However, the observations in the present study not only connect the GWAS/candidate gene COPD studies to the smoking disordered biology of airway BC and potentially to the earliest lung histologic abnormalities in cigarette smokers, but also suggest a new paradigm regarding the relationship between genetic variation and the risk for smoking-induced lung disease, at least for the 19q13.2 locus, suggesting multiple levels of genetic influences modulating the risk of COPD in smokers.

First, the data suggests that the identification of 19q13.2 as a risk locus for COPD may be relevant to disordered biology of not a single gene, but rather groups of genes clustered in specific regions of the genome and that are normally under a tight regulatory control. Consistent with this concept, not only have GWAS and candidate gene studies implicated 4 of the 13 BC smoking dysregulated genes (NFKBIB, LTBP4, EGLN2 and TGFB1) localized to 19q13.2, but almost all of the other 9 of the 13 BC smoking-dysregulated genes on 19q13.2 are associated with evidence that they also are relevant to the pathogenesis of COPD, and in some cases, lung cancer, a smoking-related disorder, for which COPD conveys a significant risk [53]. Further, the significant correlation of the expression of the 13 BC smoking up-regulated genes in nonsmokers, but less so in smokers hints toward a hypothesis of “lack of coordinate control”, in which the BC smoking dysregulated genes localized to chromosomal band 19q13.2 normally have a strong pattern of co-expression, but this is partially lost with the stress of smoking.

Second, the data also suggests that one reason why 19q13.2 is a risk locus for smoking-related development of COPD is that smoking dysregulates gene expression in airway epithelial BC, with a disproportionate fraction of these genes localized to 19q13.2. Given the critical role BC play as a the stem/progenitor cells of the airway epithelium, and that BC show the first lung histologic abnormalities associated with smoking [8], this may be the “soil” upon which the genetic variation conferring risk for COPD may function.

Together, these data provide new insights into the pathogenesis of smoking-associated chronic lung disorders, and suggest paradigms to consider regarding the links between genetic variation and the risk for smoking-induced lung disease. While all of the subjects in our study of BC were “healthy” by clinical criteria (symptoms, lung function, chest imaging), the smokers were “unhealthy” at the biologic level, with marked dysregulation of the biology of their airway BC, the stem/progenitor cells of the airway epithelium. Importantly, this dysregulated biology includes a discrete region of the genome recognized by many studies as a region associated with risk for COPD, relating genetic variability to airway BC, the cell population implicated in the development of the earliest morphologic abnormalities associated with smoking [8]. Whereas the conceptualization of the pathogenesis of COPD has been built on smoking inducing the expression of mediators such as proteases and oxidants, or the suppression of defenses such as antiproteases, antioxidants and innate immunity [54], the data in the present study not only relates genetic variability to a specific cell population central to the maintenance of airway structure and function, but it suggests there may be genetic control of the airway epithelium by smoking, and that at least one of the early events in the pathogenesis of COPD may be a loss of coordinate control of genes that are the targets of cigarette smoke. It is unknown whether this is through the effect of cigarette smoking on a single transcription factor or other controlling element region, or more likely through the effect of different components of cigarette smoking on multiple controlling regions of the BC smoking-dysregulated genes. It is known that only a fraction of smokers develop COPD. The finding that smoker BC, and not the complete airway epithelium, are vulnerable to the effects of cigarette smoke at a locus associated with both smoking and COPD supports the hypothesis that airway BC are key players in the pathogenesis of smoking-related lung disease and presents new targets to consider in developing drugs to protect the lung from the stress of smoking for individuals at risk for developing COPD.

Finally, whereas the data in the present study ties the 19q13.2 COPD risk locus to dysregulation of gene expression in BC, there are several other COPD risk loci not linked to BC [10]–[16]. In the context that dysregulation of BC biology is likely only part of the pathogenesis of COPD, there may be other COPD risk loci relevant in other cell populations central to the pathogenesis of COPD, such as pulmonary capillary endothelium and inflammatory and immune cells [54].

## Supporting Information

File S1 Supplemental Methods; Table S1. Significant Linkage Analysis, Candidate Gene and Genome-wide Association Studies Relevant to 19q13.2 as a Risk Locus for COPD; Table S2. Significant Basal Cell Smoking-responsive Genes by Category; Table S3. Over-representation Pathway Analysis Smoker Basal Cell Genes; Table S4. Demographics; Figure S1. GO Cellular Process; Figure S2. FPKM Threshold above Background.
(PDF)
