# Supplementary material for: Smoking Dysregulates the Human Airway Basal Cell Transcriptome at COPD Risk Locus 19q13.2
Source: PLoS One. 2014 Feb 3;9(2):e88051. doi: 10.1371/journal.pone.0088051 (PMC3912203; doi:10.1371/journal.pone.0088051)
Supplement: File S1 — Supplemental Methods; Table S1. Significant Linkage Analysis, Candidate Gene and Genome-wide Association Studies Relevant to 19q13.2 as a Risk Locus for COPD; Table S2. Significant Basal Cell Smoking-responsive Genes by Category; Table S3. Over-representation Pathway Analysis Smoker Basal Cell Genes; Table S4. Demographics; Figure S1. GO Cellular Process; Figure S2. FPKM Threshold above Background. (PDF) [file pone.0088051.s001.pdf]

Supplemental Methods

Sampling Airway Epithelium

Healthy nonsmokers (n=10) and healthy smokers (n=7) were evaluated at the Department of Genetic Medicine Clinical Research Facility and the Weill Cornell NIH Clinical Translational Science Center (CTSC) or the Rockefeller University CTSC using Institutional Review Board-approved clinical protocols. Nonsmoker status was confirmed using urinary nicotine (<2 ng/ml) and cotinine (<5 ng/ml) levels. All individuals had normal physical examination, pulmonary function tests and chest radiology (Table S3). The demographics of the nonsmokers and smokers were similar for gender, age and ancestry. Following informed written consent, subjects underwent flexible bronchoscopy to obtain large airway epithelium brushings as previously described [1]. Cells were detached from the brush by flicking into 5 ml of ice-cold Bronchial Epithelium Basal Medium (MEGB, Lonza, Basel, Switzerland). An aliquot of 0.5 ml was used for differential cell count. The remainder (4.5 ml) was processed immediately for basal cell purification followed by RNA extraction. The number of cells recovered by brushing was determined by counting on a hemocytometer. To quantify the percentage of epithelial and inflammatory cells and the proportions of basal, ciliated, secretory and intermediate cells recovered, cells were prepared by centrifugation (cytospin 11, Shandon Instruments, Pittsburgh, PA) and stained with Diff-Quik (Baxter Health-care, Miami, FL). In all samples, epithelial cells represented > 97% of the cell population; the proportions of epithelial cells were as previously reported [1].

Human Airway Basal Cells

The basal cells were collected and purified as described by Hackett et al [2]. Airway epithelial cells collected by brushing were pelleted by centrifugation (250 x *g*, 5 min) and disaggregated by resuspension in 0.05% trypsin-ethylenediaminetetraacetic acid (EDTA) for 5 min, at 37°C. Trypsinization was stopped by addition of HEPES buffered saline (Lonza, Basel, Switzer-

land), supplemented with 15% fetal bovine serum (FBS; GIBCO-Invitrogen, Carlsbad, CA), and the cells were again pelleted at 250 x *g*, 5 min. The pellet was resuspended with 5 ml of phosphate buffered saline, pH 7.4 (PBS), at 23°C, then centrifuged at 250 x *g*, 5 min. Following centrifugation, the PBS was removed, the cells resuspended in 5 ml of BEGM and 5 x 10^5 cells were cultured in T25 flasks in BEGM (Lonza, Basel, Switzerland), supplemented with growth factors according to the manufacturer's instructions. The antibiotics supplied by the manufacturer were replaced with gentamicin (50 µg/ml; Sigma, St Louis, MO), amphotericin B (1.25 µg/ml; Invitrogen, Carlsbad, CA), and penicillin-streptomycin (50 µg/ml; Invitrogen, Carlsbad, CA). Cultures were maintained in a humidified atmosphere of 5% CO_2, 37°C. Unattached cells were removed by changing the medium after 12 hr. Thereafter, media was changed every 2 days until time of harvest at day 7, when the cells had reached 70 to 80% confluence. The basal cells were trypsinized and cytospin slides prepared for characterization by immunohistochemistry using cell-type specific markers, including: cytokeratin 5 (basal cell; 1/50; Thermo Scientific, Rockford, IL); p63 (basal cell; 1/50; Santa Cruz Biotechnology, Inc., Santa Cruz, CA); CD151 (basal cell; 1/200; Leica Microsystems, Inc., Bannockburn, IL); N-cadherin (mesenchymal cell; 1/2500; Invitrogen, Carlsbad, CA); mucin 5AC (secretory cell; 1/50; Vector Laboratories, Burlingame, CA); TFF3 (secretory cell; 1/1000; Santa Cruz Biotechnology, Santa Cruz, CA); β-tubulin IV (ciliated cell; 1/2000 dilution; Biogenex, San Ramon, CA); chromogranin A (neuroendocrine cell; 1/5000; Thermo Scientific, Rockford, IL) and CGRP (neuroendocrine cell; 1/500; Sigma, St Loius, MO).

Sample Preparation and Sequencing

Total RNA from harvested nonsmoker and smoker basal cells was extracted with TRIzol (Invitrogen, Carlsbad, CA) with subsequent RNA clean-up using the RNeasy MinElute RNA purification kit (Qiagen, Valencia, CA) to remove residual DNA. RNA integrity was assessed using

the Agilent Technologies 2100 Bioanalyzer (Santa Clara, CA), and the NanoDrop ND-1000 spectrophotometer (NanoDrop Technologies, Wilmington, DE) to determine the RNA concentration. Samples were stored in RNAsecure (Ambion, Austin, TX) at -80°C until further analysis. The TruSeq RNA Sample Prep Kit (Illumina, San Diego, CA) was utilized as per protocol to generate poly(A)$^+$ mRNA libraries from total RNA incorporating Sera-mag magnetic oligo(dT) beads. An RNA fragmentation kit (Illumina, San Diego, CA) was used to fragment the mRNA. Random hexamer primers were next utilized to synthesize first and second strand cDNA. An "end repair" reaction was performed to blunt the fragment ends using Klenow polymerase and T4 DNA polymerase, and 3'-5' exo-nuclease was used to create the 3' adenine overhanging tail, allowing ligation of amplification adapters. Ligation products were then separated on a 2% tris-acetate EDTA -agarose gel for size selection, with subsequent purification using a QIA-quick gel extraction kit (Qiagen). These purified ligation products were PCR amplified with complementary primers and the resultant cDNA was purified with QIA-quick PCR purification kit (Qiagen, Valencia, CA), and the concentration measured by the NanoDrop spectrophotometer. Samples were then loaded onto an Illumina flowcell for paired-end 101 x 2 sequencing reactions using the Illumina HiSeq 2000, using a single flow cell with 4 samples multiplexed per lane of flow cell. Insert size was 120-210 bases.

Data Filtering, Read Mapping and Quantification of Gene Expression

HiSeq Control Software (Illumina) was used to perform image analysis, base calling and sequence analysis on HiSeq generated sequence images. Expression analysis was performed using Bowtie (v0.12.8.0, http://bowtie-bio.sourceforge.net), Tophat (v2.0.4, http://tophat.cbcb.umd.edu) and Cufflinks (v2.0.2, http://cufflinks.cbcb.umd.edu). To correct for transcript length and depth of coverage, raw paired-end sequenced reads were converted into fragments per kilobase of exon per million fragments sequenced (FPKM). Resultant fragments

were aligned (mapped) to the reference genome build UCSC hg19 using Bowtie. Remaining non-aligned reads were segmented using Tophat and re-aligned to the genome, aligning reads that span introns and determining junction splice sites. Cufflinks assembled reads into transcripts and assembled reads merged using Cuffmerge [3]; the reads generated from a transcript are directly proportional to the transcript's relative abundance. For the analysis, Tophat (which automatically runs Bowtie) was run for each read pair in a given lane. For each read pair, Tophat produced one file which contained all reads mapped to the human_g1k_v37 genome and RefSeq (http://hgdownload.cse.ucsc.edu/goldenPath/hg19/database/refGene.txt.gz) transcriptome. This process was carried out independently for each sample lane. Before running Cufflinks, Samtools (v0.1.18.0, http://samtools.sourceforge.net) was used to merge each sample's independent Tophat results into one file. This file contains all the aligned reads for each read pair. Finally, Cufflinks was run on the merged Tophat output to produce a file with FPKM values for each sample.

Supplemental References

1. Hackett NR, Heguy A, Harvey BG, O'Connor TP, Luettich K et al. (2003) Variability of antioxidant-related gene expression in the airway epithelium of cigarette smokers. Am J Respir Cell Mol Biol 29: 331-343.

2. Hackett NR, Shaykhiev R, Walters MS, Wang R, Zwick RK et al. (2011) The human airway epithelial basal cell transcriptome. PLoS One 6: e18378.

3. Trapnell C, Roberts A, Goff L, Pertea G, Kim D et al. (2012) Differential gene and transcript expression analysis of RNA-seq experiments with TopHat and Cufflinks. Nat Protoc 7: 562-578.

4. Silverman EK, Palmer LJ, Mosley JD, Barth M, Senter JM et al. (2002) Genomewide linkage analysis of quantitative spirometric phenotypes in severe early-onset chronic obstructive pulmonary disease. Am J Hum Genet 70: 1229-1239.

5. Silverman EK, Mosley JD, Palmer LJ, Barth M, Senter JM et al. (2002) Genome-wide linkage analysis of severe, early-onset chronic obstructive pulmonary disease: airflow obstruction and chronic bronchitis phenotypes. Hum Mol Genet 11: 623-632.

6. Celedon JC, Lange C, Raby BA, Litonjua AA, Palmer LJ et al. (2004) The transforming growth factor-beta1 (TGFB1) gene is associated with chronic obstructive pulmonary disease (COPD). Hum Mol Genet 13: 1649-1656.

7. Bakke PS, Zhu G, Gulsvik A, Kong X, Agusti AG et al. (2011) Candidate genes for COPD in two large data sets. Eur Respir J 37: 255-263.

8. Demeo DL, Hersh CP, Hoffman EA, Litonjua AA, Lazarus R et al. (2007) Genetic determinants of emphysema distribution in the national emphysema treatment trial. Am J Respir Crit Care Med 176: 42-48.

9. Hersh CP, Demeo DL, Lazarus R, Celedon JC, Raby BA et al. (2006) Genetic association analysis of functional impairment in chronic obstructive pulmonary disease. Am J Respir Crit Care Med 173: 977-984.

10. Smolonska J, Wijmenga C, Postma DS, Boezen HM (2009) Meta-analyses on suspected chronic obstructive pulmonary disease genes: a summary of 20 years' research. Am J Respir Crit Care Med 180: 618-631.

11. Kim WJ, Hoffman E, Reilly J, Hersh C, Demeo D et al. (2011) Association of COPD candidate genes with computed tomography emphysema and airway phenotypes in severe COPD. Eur Respir J 37: 39-43.

12. van Diemen CC, Postma DS, Aulchenko YS, Snijders PJ, Oostra BA et al. (2010) Novel strategy to identify genetic risk factors for COPD severity: a genetic isolate. Eur Respir J 35: 768-775.

13. van Diemen CC, Postma DS, Vonk JM, Bruinenberg M, Nolte IM et al. (2006) Decorin and TGF-beta1 polymorphisms and development of COPD in a general population. Respir Res 7: 89.

14. Liu DS, Li XO, Ying BW, Chen L, Wang T et al. (2010) Effects of single nucleotide polymorphisms 869 T/C and 915 G/C in the exon 1 locus of transforming growth factor-beta1 gene on chronic obstructive pulmonary disease susceptibility in Chinese. Chin Med J (Engl) 123: 390-394.

15. Ito M, Hanaoka M, Droma Y, Hatayama O, Sato E et al. (2008) The association of transforming growth factor beta 1 gene polymorphisms with the emphysema phenotype of COPD in Japanese. Intern Med 47: 1387-1394.

16. Cho MH, Washko GR, Hoffmann TJ, Criner GJ, Hoffman EA et al. (2010) Cluster analysis in severe emphysema subjects using phenotype and genotype data: an exploratory investigation. Respir Res 11: 30.

17. Su ZG, Wen FQ, Feng YL, Xiao M, Wu XL (2005) Transforming growth factor-beta1 gene polymorphisms associated with chronic obstructive pulmonary disease in Chinese population. Acta Pharmacol Sin 26: 714-720.

18. Wu L, Chau J, Young RP, Pokorny V, Mills GD et al. (2004) Transforming growth factor-beta1 genotype and susceptibility to chronic obstructive pulmonary disease. Thorax 59: 126-129.

19. Cho MH, Castaldi PJ, Wan ES, Siedlinski M, Hersh CP et al. (2012) A genome-wide association study of COPD identifies a susceptibility locus on chromosome 19q13. Hum Mol Genet 21: 947-957.

20. Hackett NR, Butler MW, Shaykhiev R, Salit J, Omberg L et al. (2012) RNA-Seq quantification of the human small airway epithelium transcriptome. BMC Genomics 13: 82.

21. Ramskold D, Wang ET, Burge CB, Sandberg R (2009) An abundance of ubiquitously expressed genes revealed by tissue transcriptome sequence data. PLoS Comput Biol 5: e1000598.

Supplemental Tables

**Table S1.**	Significant Linkage Analysis, Candidate Gene and Genome-wide Association Studies Relevant to 19q13.2 as a Risk Locus for COPD
**Table S2.**	Significant Basal Cell Smoking-responsive Genes by Category
**Table S3.**	Over-representation Pathway Analysis Smoker Basal Cell Genes
**Table S4.**	Demographics

Supplemental Figures

**Figure S1**	GO Cellular Process
**Figure S2**	FPKM Threshold above Background

Table S1. Significant Linkage Analysis, Candidate Gene and Genome-wide Association Studies Relevant to 19q13.2 as a Risk Locus for COPD

Type of study	Chr locus	Gene	SNP[1] / risk allele	Reference
Linkage analysis	Chr19	N/A[1]	N/A	[4,5]
	19q	N/A	N/A	[6]
Candidate gene	19q13.2	NFKBIB	rs2241704, rs2053071	[7]
		LTBP4	rs2077407	[8]
			rs2077407, rs2303729, rs1131620, rs1051303	[9]
		TGFB1	rs1982073, rs1800469, rs2241712	[8]
			rs1800469, rs2241712, rs1982073	[9]
			rs2241712, rs1800469, rs1982073	[6]
			rs2241712, rs1800469, rs1800470	[10]
			rs6957	
			rs1800469, rs1800470, rs1800471, rs11083616, rs11466321	[11]
			rs1800469, rs1982073, rs6957	[12]
			rs1800469, rs1982073, rs6957	[13]
			869T/C	[14]
			rs1800469, rs1982073	[15]
			rs1800470	[16]
			800A / 509C	[17]
			rs1982073	[18]
GWAS	19q13.2	EGLN2	rs7937, rs2604894[2]	[19]

[1] SNP - single nucleotide polymorphism; NA = not applicable.
[2] SNPs are either near EGLN2 or EGLN2 is the nearest gene to this identified SNP.

Table S2. Significant Basal Cell Smoking-responsive Genes by Functional Category[1]

Functional category	Classification	Gene symbol	Gene title	Nonsmoker mean expression level (FPKM)[2]	Smoker mean expression level (FPKM)[2]	Smoker *vs* nonsmoker: Absolute difference[3]	Smoker *vs* nonsmoker: Fold-change[4]	Smoker *vs* nonsmoker: p value[5]
Transcription factors	Basic leucine zipper	JUNB	jun B proto-oncogene	44.6	134.2	89.5	3.0	0.004
		CEBPD	CCAAT/enhancer binding protein (C/EBP), delta	25.4	71.0	45.6	2.8	0.046
		CEBPB	CCAAT/enhancer binding protein (C/EBP), beta	26.4	55.3	28.9	2.1	0.021
		JUND	jun D proto-oncogene	14.4	42.7	28.3	3.0	0.006
		MAFK	v-maf musculoaponeurotic fibrosarcoma oncogene homolog K (avian)	5.8	15.0	9.2	2.6	0.003
	Beta-scaffold	SOX15	SRY (sex determining region Y)-box 15	41.1	64.8	23.7	1.6	0.004
		TCF3	transcription factor 3 (E2A immunoglobulin enhancer binding factors E12/E47)	16.7	28.8	12.1	1.7	0.001
		NFKB2	nuclear factor of kappa light polypeptide gene enhancer in B-cells 2 (p49/p100)	14.0	21.7	7.7	1.6	0.026
		TBX1	T-box 1	5.1	9.2	4.1	1.8	0.016
		RELB	v-rel reticuloendotheliosis viral oncogene homolog B	4.5	8.1	3.6	1.8	0.035
		TAF1C	TATA box binding protein (TBP)-associated factor, RNA polymerase I, C, 110kDa	4.1	6.9	2.8	1.7	0.009
		BRF1	BRF1 homolog, subunit of RNA polymerase III transcription initiation factor IIIB (S. cerevisiae)	3.7	5.8	2.2	1.6	0.016
		BANP	BTG3 associated nuclear protein	2.7	4.6	1.8	1.7	0.004
	Helix-turn-helix	HSF1	heat shock transcription factor 1	28.4	50.5	22.1	1.8	0.004
		PITX1	paired-like homeodomain 1	9.6	30.8	21.3	3.2	0.001
		TWIST2	twist homolog 2 (Drosophila)	1.2	2.9	1.6	2.3	0.034
		MYPOP	Myb-related transcription factor, partner of profilin	1.6	2.5	0.9	1.5	0.046
		HSF4	heat shock transcription factor 4	0.3	1.1	0.7	3.4	0.013
	Zinc finger	SLC2A4RG	SLC2A4 regulator	22.3	37.9	15.6	1.7	0.034
		NR2F6	nuclear receptor subfamily 2, group F, member 6	22.1	36.3	14.3	1.6	0.007
		SOLH	small optic lobes homolog (Drosophila)	3.5	14.5	11.0	4.1	0.001
		KLF16	Kruppel-like factor 16	8.8	18.6	9.7	2.1	0.001
		ZNF358	zinc finger protein 358	6.5	13.3	6.8	2.1	0.002
		ZBTB7A	zinc finger and BTB domain containing 7A	10.1	16.9	6.8	1.7	0.005
		ZNF213	zinc finger protein 213	3.9	8.9	5.0	2.3	0.020
		MZF1	myeloid zinc finger 1	1.6	5.3	3.7	3.4	0.013
		E4F1	E4F transcription factor 1	3.5	7.2	3.7	2.1	0.001
		ZNF444	zinc finger protein 444	3.0	5.9	2.9	2.0	0.001
		ZNF668	zinc finger protein 668	1.2	3.1	1.8	2.5	0.001
		FIZ1	FLT3-interacting zinc finger 1	2.1	3.4	1.3	1.6	0.001
		ZNF775	zinc finger protein 775	0.6	1.7	1.0	2.6	0.001

Table S2. Significant Basal Cell Smoking-responsive Genes by Category[1] (cont., page 2)

Functional category	Classification	Gene symbol	Gene title	Nonsmoker mean expression level (FPKM)[2]	Smoker mean expression level (FPKM)[2]	Smoker *vs* nonsmoker: Absolute difference[3]	Smoker *vs* nonsmoker: Fold-change[4]	Smoker *vs* nonsmoker: p value[5]
	Other	MLLT1	myeloid/lymphoid or mixed-lineage leukemia (trithorax homolog, Drosophila); translocated to, 1	11.7	19.1	7.5	1.6	0.001
		HCFC1	host cell factor C1 (VP16-accessory protein)	8.2	13.0	4.8	1.6	0.030
		MKL1	megakaryoblastic leukemia (translocation) 1	9.0	13.6	4.6	1.5	0.003
		GMEB2	glucocorticoid modulatory element binding protein 2	4.8	8.8	4.0	1.8	0.026
		ARID3A	AT rich interactive domain 3A (BRIGHT-like)	2.1	3.9	1.8	1.9	0.007
		DMRTA2	DMRT-like family A2	1.3	2.4	1.1	1.8	0.038
		IER2	immediate early response 2	38.0	68.6	30.6	1.8	0.008
		CIC	capicua homolog (Drosophila)	8.5	14.9	6.4	1.8	0.015
Adhesion molecules		LAMA5	laminin, alpha 5	19.7	54.6	34.8	2.8	0.002
		AMIGO3	adhesion molecule with Ig-like domain 3	0.3	0.7	0.4	2.3	0.030
		FMNL1	formin-like 1	1.0	1.8	0.8	1.7	0.049
		MEGF8	multiple EGF-like-domains 8	3.8	6.5	2.7	1.7	0.006
		PCDHGB5	protocadherin gamma subfamily B, 5	0.6	1.1	0.5	1.7	0.030
		ADRM1	adhesion regulating molecule 1	51.6	85.6	34.0	1.7	0.013
		ITGB4	integrin, beta 4	233.5	375.7	142.2	1.6	0.032
Cytoskeletal associated proteins		MIB2	mindbomb homolog 2 (Drosophila)	2.0	9.3	7.3	4.6	0.001
		EPPK1	epiplakin 1	6.5	21.5	15.0	3.3	0.002
		PLEKHH3	pleckstrin homology domain containing, family H (with MyTH4 domain) member 3	5.2	12.3	7.1	2.4	0.016
		GAS2L1	growth arrest-specific 2 like 1	4.6	10.5	5.8	2.3	0.031
		SHANK3	SH3 and multiple ankyrin repeat domains 3	1.0	1.9	0.9	2.0	0.003
		KLHL17	kelch-like 17 (Drosophila)	1.3	2.6	1.3	2.0	0.014
		MID1IP1	MID1 interacting protein 1 (gastrulation specific G12 homolog (zebrafish))	26.4	48.3	21.9	1.8	0.032
		PLXNA3	plexin A3	5.0	9.1	4.1	1.8	0.006
		KLHL26	kelch-like 26 (Drosophila)	1.6	2.9	1.3	1.8	0.044
		ANTXR2	anthrax toxin receptor 2	18.3	33.4	15.1	1.8	0.006
		AZI1	5-azacytidine induced 1	1.6	2.7	1.2	1.7	0.039
		DBN1	drebrin 1	30.9	53.0	22.2	1.7	0.003
		FLNC	filamin C, gamma	0.1	0.2	0.1	1.7	0.027
		HIP1R	huntingtin interacting protein 1 related	8.2	13.4	5.2	1.6	0.005
		ARPC1B	actin related protein 2/3 complex, subunit 1B, 41kDa	114.8	180.7	65.9	1.6	0.012
		CDC42EP1	CDC42 effector protein (Rho GTPase binding) 1	20.5	42.8	22.3	2.1	0.033

Table S2. Significant Basal Cell Smoking-responsive Genes by Category[1] (cont., page 3)

Functional category	Classification	Gene symbol	Gene title	Nonsmoker mean expression level (FPKM)[2]	Smoker mean expression level (FPKM)[2]	Smoker *vs* nonsmoker: Absolute difference[3]	Smoker *vs* nonsmoker: Fold-change[4]	Smoker *vs* nonsmoker: p value[5]
		MICAL1	microtubule associated monoxygenase, calponin and LIM domain containing 1	7.9	13.7	5.8	1.7	0.041
		TUBGCP6	tubulin, gamma complex associated protein 6	3.5	5.4	1.9	1.5	0.012
		MAP1S	microtubule-associated protein 1S	3.3	13.7	10.4	4.1	0.001
		HOOK2	hook homolog 2 (Drosophila)	8.9	15.7	6.8	1.8	0.003
		PAK4	p21 protein (Cdc42/Rac)-activated kinase 4	9.3	15.3	6.0	1.7	0.024
Extracellular matrix proteins		AGRN	agrin	40.9	116.6	75.7	2.9	0.001
		LTBP4	latent transforming growth factor beta binding protein 4	10.4	26.8	16.4	2.6	0.003
		EGFL7	EGF-like-domain, multiple 7	3.6	8.8	5.2	2.4	0.004
		HSPG2	heparan sulfate proteoglycan 2	19.0	37.6	18.6	2.0	0.011
		COL18A1	collagen, type XVIII, alpha 1	6.2	12.3	6.1	2.0	0.023
		COL16A1	collagen, type XVI, alpha 1	17.0	33.1	16.1	1.9	0.015
		COL7A1	collagen, type VII, alpha 1	63.2	122.6	59.4	1.9	0.026
		FSTL3	follistatin-like 3 (secreted glycoprotein)	46.5	75.4	28.9	1.6	0.004
Receptors	G-protein coupled receptors	MC1R	melanocortin 1 receptor (alpha melanocyte stimulating hormone receptor)	0.5	1.0	0.4	1.9	0.023
		GPR157	G protein-coupled receptor 157	2.8	5.1	2.3	1.8	0.019
		CELSR1	cadherin, EGF LAG seven-pass G-type receptor 1 (flamingo homolog, Drosophila)	18.0	31.0	13.0	1.7	0.018
	Ligand-dependent nuclear receptors	NR1H2	nuclear receptor subfamily 1, group H, member 2	13.8	25.1	11.3	1.8	0.014
		NR2F6	nuclear receptor subfamily 2, group F, member 6	22.1	36.3	14.3	1.6	0.007
		ESRRA	estrogen-related receptor alpha	18.7	28.1	9.4	1.5	0.011
		AR	androgen receptor	0.7	0.3	0.4	-2.2	0.039
	Transmembrane receptors	SCARF2	scavenger receptor class F, member 2	0.2	0.9	0.7	4.8	0.006
		TSPO	translocator protein (18kDa)	92.7	220.8	128.1	2.4	0.001
		SIGIRR	single immunoglobulin and toll-interleukin 1 receptor (TIR) domain	5.7	12.7	7.0	2.2	0.030
		TNFRSF25	tumor necrosis factor receptor superfamily, member 25	5.3	11.1	5.7	2.1	0.013
		GPC1	glypican 1	42.4	86.0	43.6	2.0	0.021
		PLXNA3	plexin A3	5.0	9.1	4.1	1.8	0.006
		PLXNB2	plexin B2	39.8	66.7	26.9	1.7	0.018
		ITGB4	integrin, beta 4	233.5	375.7	142.2	1.6	0.032

Table S2. Significant Basal Cell Smoking-responsive Genes by Category[1] (cont., page 4)

Functional category	Classification	Gene symbol	Gene title	Nonsmoker mean expression level (FPKM)[2]	Smoker mean expression level (FPKM)[2]	Smoker *vs* nonsmoker: Absolute difference[3]	Smoker *vs* nonsmoker: Fold-change[4]	Smoker *vs* nonsmoker: p value[5]
		FGFRL1	fibroblast growth factor receptor-like 1	9.3	14.8	5.5	1.6	0.005
		PLXNA1	plexin A1	20.7	32.8	12.1	1.6	0.025
		LRP10	low density lipoprotein receptor-related protein 10	56.1	87.3	31.2	1.6	0.022
Growth factors		RABEP2	rabaptin, RAB GTPase binding effector protein 2	3.3	6.0	2.7	1.8	0.033
		TGFB1	transforming growth factor, beta 1	36.1	61.3	25.2	1.7	0.002
Ion channels	Mechanosensitive ion channels	PIEZO1	piezo-type mechanosensitive ion channel component 1	16.0	33.2	17.3	2.1	0.001
	Calcium ion channels	TRPV3	transient receptor potential cation channel, subfamily V, member 3	1.0	3.6	2.6	3.6	0.026
		PKD1	polycystic kidney disease 1 (autosomal dominant)	4.5	16.1	11.6	3.5	0.001
		ORAI1	ORAI calcium release-activated calcium modulator 1	9.4	15.5	6.1	1.6	0.016
	Chloride ion channels	CLCN7	chloride channel 7	6.5	12.7	6.2	1.9	0.006

[1] Significant basal cell smoking responsive genes in a selection of relevant categories.
[2] FPKM = fragments per kilobase of exon per million fragments mapped.
[3] Absolute difference = smoker mean-nonsmoker mean.
[4] Fold-change = mean in smokers/mean in nonsmokers.
[5] False discovery rate controlled to 0.05 using Partek ‘step-up’ (Benjamini-Hochberg) procedure.

Table S3. Consensus Pathway Database: Over-representation Gene Set Analysis of 676 Smoking Dysregulated Human Airway Basal Cell Genes[1]

Pathway	Pathway source	p-value	q-value[2]	Our genes / effective genes set (%)	Effective gene set[3]	Total gene set[4]	Genes
Integrin	INOH[5]	0.002	0.375	10/85 (11.7%)	85	125	ITGB4; GPC1; COL18A1; HSPG2; DOCK6; AGRN; MAP2K2; COL16A1; BCAR1; COL7A1
RAC1 signaling pathway	PID[6]	0.003	0.375	7/47 (14.9%)	47	54	PIP5K1C; ARPC1B; MAP3K11; CYBA; MAP2K7; ARHGDIA; BCAR1
Regulation of nuclear SMAD2/3 signaling	PID[6]	0.003	0.375	8/61 (13.1%)	61	78	CEBPB; ZBTB17; TCF3; AR; PI-AS4; CTBP1; SIN3B; CREBBP
Notch-mediated HES/HEY network	PID[6]	0.004	0.375	6/38 (15.7%)	38	48	TCF3; NOTCH1; AR; CTBP1; NCOR2; CREBBP
HS-GAG degradation	Reactome	0.004	0.375	4/17 (23.5%)	17	20	AGRN; GPC1; HSPG2; IDUA
Notch Signaling Pathway	Wikipathways	0.005	0.375	6/39 (15.4%)	39	47	NOTCH1; DVL1; CTBP1; NCOR2; CREBBP; NUMBL
A tetrasaccharide linker sequence is required for GAG synthesis	Reactome	0.005	0.375	4/18 (22.2%)	18	26	AGRN; GPC1; B3GALT6; HSPG2
Regulation of toll-like receptor signaling pathway	Wikipathways	0.006	0.375	10/96 (10.4%)	96	147	TICAM1; SOCS1; SIGIRR; TOLLIP; MAP2K2; MAP2K7; RBCK1; NFKB2; IRAK1; FBXW5
Notch signaling pathway - Homo sapiens (human)	KEGG[7]	0.006	0.375	6/41 (14.6%)	41	47	NOTCH1; DVL1; CTBP1; NCOR2; CREBBP; NUMBL
EBV LMP1 signaling	Wikipathways	0.007	0.375	4/19 (21.0%)	19	22	IRAK1; NFKB2; PDLIM7; TRADD
Sulfur relay system - Homo sapiens (human)	KEGG[7]	0.007	0.375	3/10 (3.0%)	10	10	CTU2; MPST; CTU1
TLR ECSIT MEKK1 p38	INOH[5]	0.008	0.379	4/20 (2.0%)	20	23	MAP2K2; IRAK1; MAP2K7; TOL-LIP

Table S3. Consensus Pathway Database: Over-representation Gene Set Analysis of 676 Smoking Dysregulated Human Airway Basal Cell Genes[1] (cont. page 2)

Pathway	Pathway source	p-value	q-value[2]	Our genes / effective genes set (%)	Effective gene set[3]	Total gene set[4]	Genes
EGFR1	NetPath	0.009	0.379	28/429 (6.5%)	429	462	CC2D1A; STK11; JUND; MAP2K2; BAD; MAP2K7; PLEKHN1; CE-BPB; PKP3; EPPK1; SLC12A7; LSR; DYRK1B; ITGB4; PTPN23; EPN1; HGS; PLEC; CTBP1; BCAR1; TNK2; SOCS1; NCK2; TOLLIP; SCRIB; CASKIN2; RBCK1; AP2A1
Rho GTPase cycle	Reactome	0.009	0.379	10/103 (9.7%)	103	127	OBSCN; PLEKHG5; ARHGEF18; ARHGDIA; PRR5; ARHGAP39; RHOT2; GMIP; RHOB; RHOD
Signaling by Rho GTPases	Reactome	0.009	0.379	10/103 (9.7%)	103	127	OBSCN; PLEKHG5; ARHGEF18; ARHGDIA; PRR5; ARHGAP39; RHOT2; GMIP; RHOB; RHOD

[1] Significant smoking dysregulated human airway basal genes (fold change>1.5, pFDR<0.05); pathways with p value <0.01 shown.
[2] Multiple test correction using false discovery rate method.
[3] Total number of genes in pathway present in all genes expressed above background (FPKM>0.04).
[4] Total number of genes in pathway.
[5] INOH - Integrating Network Objects with Hierarchies.
[6] PID - Pathway Interaction Database.
[7] KEGG - Kyoto Encyclopedia of Genes and Genomes.

Table S4. Demographics

Parameter	Nonsmokers	Smokers
n	10	7
Gender (male/female)	10/0	6/1
Age, yr (mean ± SD)	38.3 ± 12.0	46.7 ± 8.6
Race B/W/H/O[1]	3/2/4/1	6/0/1/0
Smoking history, pack-yr	N/A	30.0 ± 10.3
Urinary nicotine, ng/ml	0	1754 ± 1038
Urinary cotinine, ng/ml	0	1516 ± 807
Venous carboxyhemoglobin, %[2]	1.3 ± 0.8	3.0 ± 1.1
Pulmonary Function Parameters[3]		
FVC	110 ± 10	115 ± 9
FEV1	103 ± 13	110 ± 5
FEV1/FVC	78 ± 4	77 ± 3
TLC	102 ± 16	97 ± 8
DLCO	94 ± 13	83 ± 4

[1] B = Black; W = White; H = Hispanic; O = Other.

[2] Carboxyhemoglobin <1.5% in nonsmokers.

[3] Pulmonary function testing parameters are given as % of predicted value with the exception of FEV1/FVC, which is reported as % observed; FVC - forced vital capacity; FEV1 - forced expiratory volume in 1 sec; TLC - total lung capacity; DLCO - diffusing capacity.

Supplemental Figure Legends

Figure S1. Hierarchical mapping of gene ontology (GO) cellular processes in the smoking dysregulated human basal cell transcriptome. The dominant processes include transmembrane receptor protein serine/threonine signaling pathway; transmembrane receptor protein tyrosine kinase signaling pathway; neuropeptide signaling pathway; G protein signaling, coupled to cyclic nucleotide second messenger; G protein signaling, coupled to IP3 second messenger phospholipase C activating; and JAK-STAT cascade.

Figure S2. Establishment of detection limit for gene expression for RNA-Seq assessment of gene expression of human airway basal cells of healthy nonsmokers. **A.** Distribution of fragments per kilobase of exon per million fragments mapped (FPKM) for exons (blue), introns (red), and intergenic regions (green). FPKM depends on the size and read numbers mapped in the region considered. The FPKM for introns and intergenic regions was calculated by selecting intronic and intergenic regions throughout the genome that match the size of the exons analyzed, i.e., the size is comparable for the introns, intergenic regions and exons. **B.** Estimate of maximum detectable level of expression (FPKM 0.04) determined from an estimate of false discovery rate (FDR) (red) and false negative rate (FNR) (purple) [20,21]. **C.** Average FPKM expression levels (log_{10}). Dashed line represents the 0.04 threshold.

Figure S1

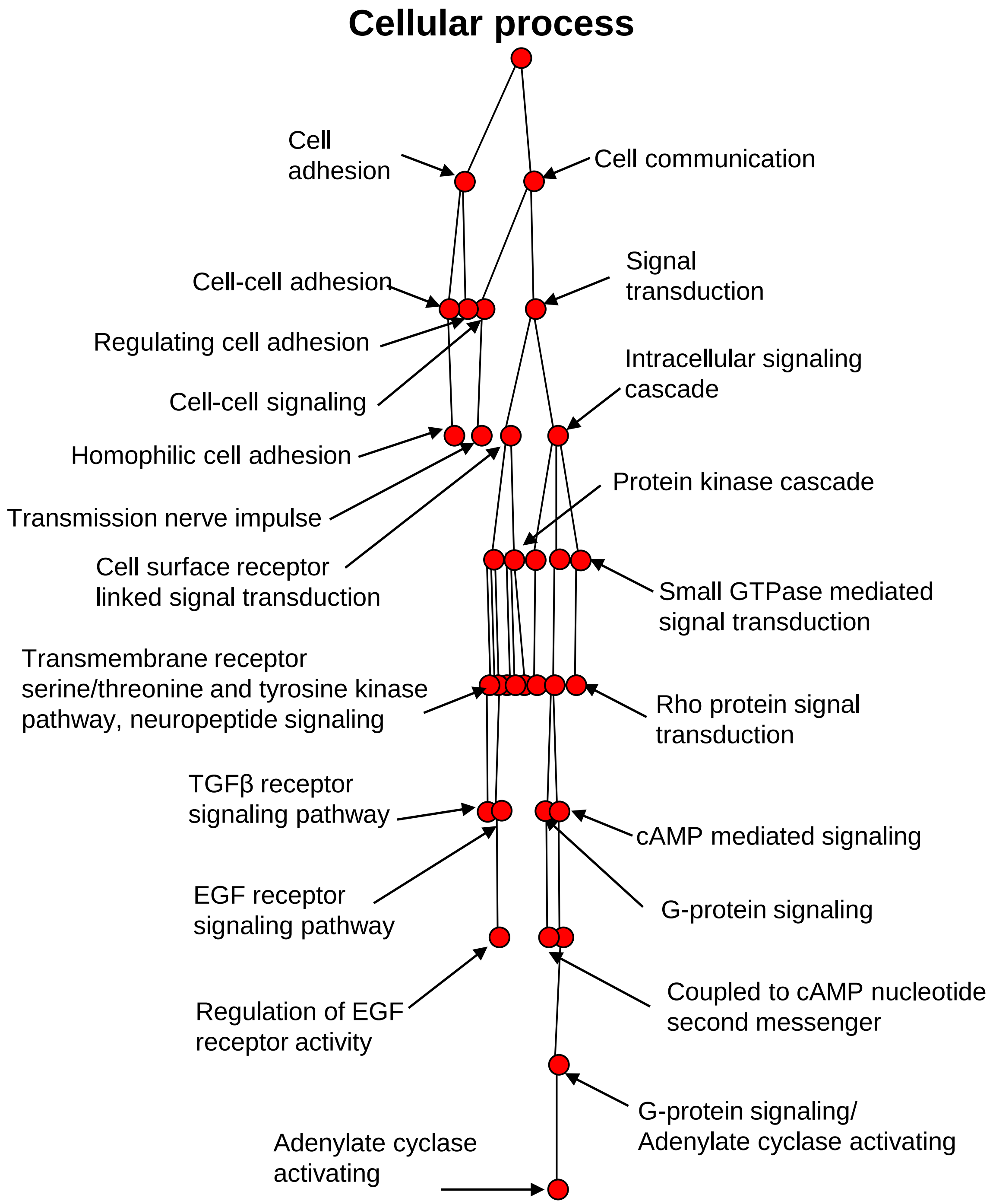
Cellular process
Cell
adhesion
Cell communication
Cell-cell adhesion
Signal
transduction
Regulating cell adhesion
Cell-cell signaling
Intracellular signaling
cascade
Homophilic cell adhesion
Transmission nerve impulse
Protein kinase cascade
Cell surface receptor
linked signal transduction
Small GTPase mediated
signal transduction
Transmembrane receptor
serine/threonine and tyrosine kinase
pathway, neuropeptide signaling
Rho protein signal
transduction
TGFβ receptor
signaling pathway
cAMP mediated signaling
EGF receptor
signaling pathway
G-protein signaling
Regulation of EGF
receptor activity
Coupled to cAMP nucleotide
second messenger
G-protein signaling/
Adenylate cyclase activating
Adenylate cyclase
activating

Figure S2

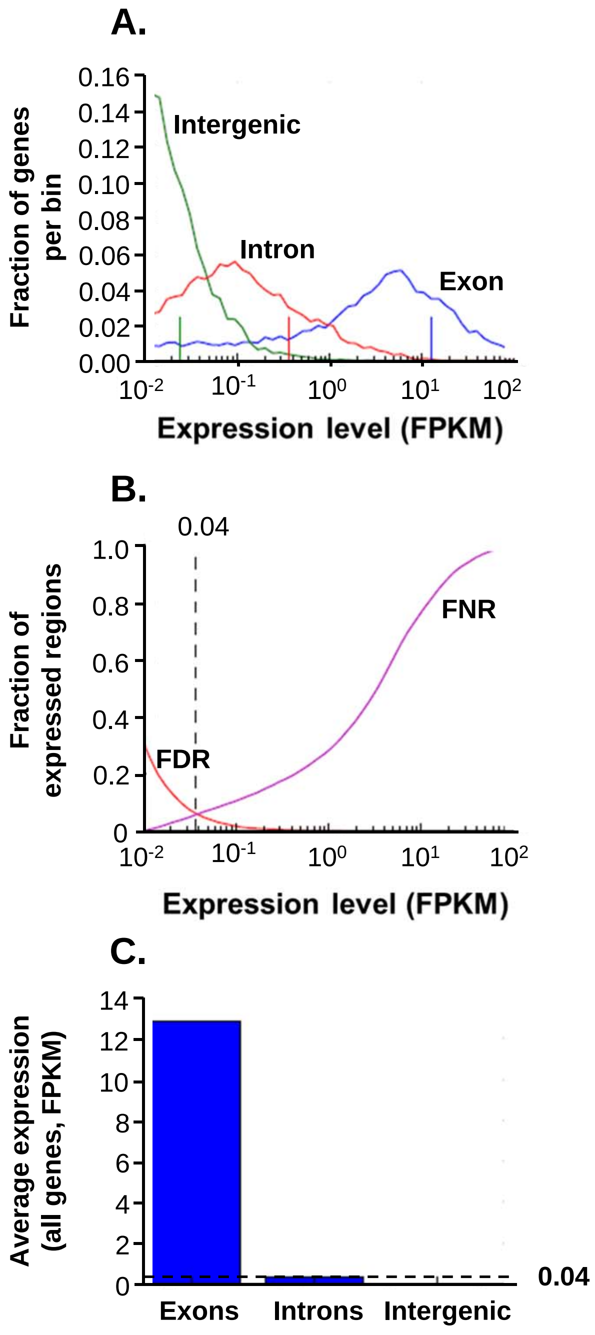
A.
Fraction of genes per bin
0.16
0.14
0.12
0.10
0.08
0.06
0.04
0.02
0.00
Intergenic
Intron
Exon
10^{-2} 10^{-1} 10^{0} 10^{1} 10^{2}
Expression level (FPKM)
B.
0.04
Fraction of expressed regions
1.0
0.8
0.6
0.4
0.2
0
FNR
FDR
10^{-2} 10^{-1} 10^{0} 10^{1} 10^{2}
Expression level (FPKM)
C.
Average expression (all genes, FPKM)
14
12
10
8
6
4
2
0
0.04
Exons
Introns
Intergenic
